# Dipeptidyl peptidase-4 inhibitor decreases the risk of atrial fibrillation in patients with type 2 diabetes: a nationwide cohort study in Taiwan

**DOI:** 10.1186/s12933-017-0640-5

**Published:** 2017-12-19

**Authors:** Chia-Yu Chang, Yung-Hsin Yeh, Yi-Hsin Chan, Jia-Rou Liu, Shang-Hung Chang, Hsin-Fu Lee, Lung-Sheng Wu, Kun-Chi Yen, Chi-Tai Kuo, Lai-Chu See

**Affiliations:** 1 College of Medicine, Chang Gung University, Taoyuan, 33302 Taiwan; 2 Department of Cardiology, Chang Gung Memorial Hospital, Linkou, Taoyuan, 33305 Taiwan; 3 Microscopy Core Laboratory, Chang Gung Memorial Hospital, Linkou, Taoyuan, 33305 Taiwan; 4 Department of Public Health, College of Medicine, Chang Gung University, No. 259, Wenhua 1st Rd., Guishan, Taoyuan, 33302 Taiwan; 5 Biostatistics Core Laboratory, Molecular Medicine Research Center, Chang Gung University, Taoyuan, 33302 Taiwan; 6 Division of Rheumatology, Allergy and Immunology, Chang Gung Memorial Hospital, Linkou, Taoyuan, 33305 Taiwan

**Keywords:** Dipeptidyl peptidase-4 inhibitor, Type 2 diabetes mellitus, Atrial fibrillation

## Abstract

**Background:**

Whether dipeptidyl peptidase-4 inhibitor (DPP4i) is associated with a lower risk of new-onset atrial fibrillation (AF) in patients with diabetes remains unclear. This study aimed to evaluate the risk of AF associated with use of DPP4i among a longitudinal cohort of patients with diabetes.

**Methods:**

Over a 3-year period, 480,000 patients with diabetes were analyzed utilizing Taiwan’s National Health Insurance Research Database and 90,880 patients taking metformin as first-line therapy were enrolled. Patients were further divided into two groups: (1) DPP4i users: those taking DPP4i and (2) non-DPP4i users: those prescribed other hypoglycemic agents (HAs) as second-line drug. Study end point was defined by diagnosis of AF, addition of any third-line HA, or the end of the study period (December 31, 2013), whichever came first.

**Results:**

A total of 16,017 DPP4i users and 74,863 non-DPP4i users were eligible for the study. For the DPP4i group, most patients were prescribed sitagliptin (n = 12,180; 76%). Among the non-DPP4i group, most patients took sulfonylurea (n = 60,606; 81%) as their second-line medication. DPP4i users were associated with a lower risk of new-onset AF compared with non-DPP4i users after propensity-score weighting (hazard ratio 0.65; *P* < 0.0001). Subgroup analysis showed that DPP4i user were associated with a lower risk of new-onset AF compared with non-DPP4i users in most subgroups. Multivariate analysis indicated that use of DPP4i was associated with lower risk of new-onset AF and age > 65 years, presence of hypertension, and ischemic heart disease were independent risk factors for new-onset AF.

**Conclusions:**

Among patients with diabetes prescribed with metformin, the patients with DPP4i as second HA were associated with a lower risk of AF compared with the patients with other drugs as second HAs in real-world practice.

**Electronic supplementary material:**

The online version of this article (10.1186/s12933-017-0640-5) contains supplementary material, which is available to authorized users.

## Introduction

Atrial fibrillation (AF) is the most common cardiac arrhythmia and significantly increases the risk of comorbidity and mortality [1, 2] with a threefold increased risk of heart failure and a fivefold increased risk of stroke [3–6]. As the world population ages, the prevalence of AF is predicted to increase by 2.5 fold in the next 50 years [7]. Diabetes mellitus (DM) is an important independent risk factor for AF [8–12]. In a previous study, AF occurred in 14.9% of diabetic patients and 10.3% in non-diabetic patients [8]. Furthermore, diabetes was highly associated with the prevalence of metabolic syndrome, which is also associated with a higher risk for AF [8]. Alogliptin, a dipeptidyl peptidase-4 inhibitor (DPP-4i), not only has anti-hyperglycemic effects, but can also inhibit the maintenance of AF induced by tachy-pacing, as shown in a recent animal study [13]. However, Only a few studies investigated if DPP4i has cardiac protective effects including AF [14–16]. This study had as its underlying hypothesis that DPP4i could potentially reduce the incidence of AF in type-2 diabetic patients. The goal of the present study was to evaluate the risk of AF associated with use of DPP4i in a nationwide cohort study of diabetic patients in Taiwan.

## Materials and methods

### Data source

This study was approved by the Institutional Review Board of Chang Gung Memorial Hospital, Linkou, Taiwan. Informed consent was waived because the original identification number of each patient in the National Health Insurance (NHI) research database (NHIRD) of Taiwan was encrypted and de-identified to protect their privacy. The NHI program is a compulsory universal health insurance program in Taiwan which provides comprehensive medical care coverage to more than 99% of Taiwanese residents. The NHIRD of the National Health Research Institutes of Taiwan included detailed health care information for 23.72 million enrollees in 2014 [17].

### Study cohort and outcomes

**Fig. 1:**
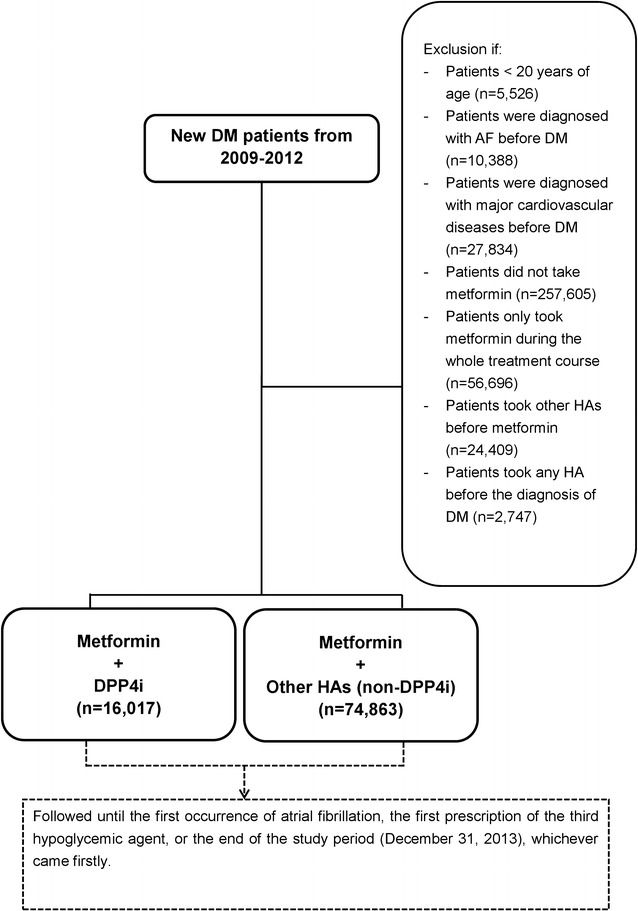
Enrollment of diabetic patients taking metformin plus DPP-4 inhibitor versus other hypoglycemic agents. A total of 16,017 diabetic patients taking metformin plus DPP-4 inhibitor are compared with 74,863 patients prescribed other hypoglycemic agents (including sulfonylurea, alpha glucosidase inhibitor, thiazolidinedione, meglitinide, insulin, or glucagon-like peptide 1). *AF* atrial fibrillation, *DM* diabetes mellitus, *DPP4i* dipeptidyl peptidase-4 inhibitor, *HA* hypoglycemic agent

From 2009 to 2012, 480,000 patients with diabetes were analyzed utilizing a Longitudinal Cohort of Diabetes Patients Database (LHDB) using newly diagnosed DM codes based on the International Classification of Diseases, ninth revision, Clinical Modification (ICD-9-CM) as previous described [18]. A flowchart of enrollment of the study cohort is summarized in Fig. 1. Subjects who were less than 20 years of age (n = 5526), had the diagnosis of AF (n = 10,388), or any cardiovascular event (n = 27,834) before the diagnosis of diabetes were excluded from the study. In Taiwan, metformin is considered a first-line hypoglycemic agent (HA) according to the current guidelines. Second-line HAs (which include sulfonylurea, alpha glucosidase inhibitor, thiazolidinedione (TZD), meglitinide, insulin, GLP-1 analogue, and DPP4i) are administered when inadequate therapeutic response to metformin is suspected, according to the current guidelines and payment criteria of the NHI in Taiwan. Subjects who were not prescribed any HA during their whole treatment course, who were first prescribed a HA other than metformin, who were only prescribed metformin during their whole treatment course, or who were prescribed with any HAs before the diagnosis of diabetes were also excluded from the current study (n = 341,457). Finally, 90,880 patients with diabetes taking metformin as the first-line therapy were enrolled in the study. Study subjects were further divided into two groups: those taking DPP4i (the DPP4i group) versus those prescribed other HAs as second-line drug (the non-DPP4i group). The DPP4i group (n = 16,017) was defined by the use of DPP4i as the second-line HA. The non-DPP4i group (n = 74,863) was defined by the use of other HAs as the second-line HA. The first claim date of the DPP4i group or the non-DPP4i group was defined as the drug index date. The study outcome was defined by the diagnosis of AF based on the ICD-9-CM code of 427.31, in either an in-patient or outpatient department at least once. The follow-up period was defined from the index date until the occurrence of the first study outcome (AF), the addition of any new HA due to inadequate sugar control, or the end of the study period (December 31, 2013), whichever came first.

### Covariates

Risk factors for cardiovascular events and use of medication at baseline were obtained from claim records using the above diagnoses or medication codes prior to the index date. A history of specific prescribed medicines was confined to medications used at least once within the 3 months preceding the index date. The ICD-9-CM codes used to identify the study outcomes and covariates are summarized in Additional file 1: Table S1.

### Statistical analysis

Propensity score method, which simulates the gold-standard of a randomized clinical trial (RCT) for observational data, was used to compare the effect between the two study groups on study outcomes. Inverse probability of treatment weighting (IPTW) of propensity scores was used to balance covariates across the two study groups [19]. The balance of potential confounders at baseline (index date) between the two study groups was evaluated by using standardized mean difference (SMD), rather than using statistical testing, because balance is a property of the sample and not of an underlying population. The value of absolute of SMD ≤ 0.1 indicates a negligible difference in potential confounders between the two study groups. Risk of study outcomes over time for the DPP-4 inhibitor group compared with non-DPP-4 inhibitor group (reference) was obtained by using survival analysis (Kaplan–Meier method for univariate analysis and Cox proportional hazards regression for multivariate analysis) after IPTW. Subgroup analysis was performed to determine whether the DPP4i group continued to have a lower risk of new-onset AF when compared with non-DPP4i in subgroups. Statistical significance was defined at a *P* value < 0.05. All statistical analyses were performed using SAS 9.3 (SAS Institute Inc., Cary, North Carolina).

## Results

A total of 16,017 DPP4i users and 74,863 non-DPP4i users were eligible for the study. Most patients in the DPP4i group were prescribed sitagliptin (n = 12,180, 76%); while 291, 1501 and 2045 patients were prescribed linagliptin (2%), saxagliptin (9%), and vildagliptin (13%), respectively. Among the non-DPP4i group, most patients were prescribed sulfonylurea (n = 60,606, 81%) as the second-line HA. In addition, 4087, 4783, 2334, 1032, and five patients were prescribed alpha glucosidase inhibitor (5%), meglitinide (6%), thiazolidinedione (3%), insulin (1%), and GLP-1 analogue (0%), respectively. There were 2016 patients (4%) taking more than two second-line HAs concurrently.

Table 1 summarizes the baseline demographic characteristics, comorbidities, and medication differences between the two groups. Before propensity score weighting, the DPP4i group had a higher use of statins and angiotensin-converting enzyme inhibitor/angiotensin receptor blockers than non-DPP4i group, while age, gender, comorbidities and other medications were all similar between two study groups at baseline (all ASMD < 0.1). After propensity-score weighting, the two study groups were well-balanced in all characteristics (all ASMD < 0.1).

**Table 1:** Baseline characteristics of diabetic patients taking metformin plus DPP4i versus other hypoglycemic agents, before and after propensity score weighting

	Before weighting			After weighting		
	DPP4i users	Non-DPP4i users	Standardized mean difference	DPP4i users	Non-DPP4i users	Standardized mean difference
	(n = 16,017)	(n = 74,863)		(n = 16,017)	(n = 74,863)	
Follow-up time (years)						
Mean ± SD	2.04 ± 1.21	2.41 ± 1.27		2.07 ± 2.90	2.41 ± 1.39	
Age at index date						
Mean ± SD	54.51 ± 12.53	54.88 ± 12.20		54.43 ± 30.16	54.88 ± 13.41	
< 65 year	80.23%	79.59%	0.0162	79.87%	79.70%	0.0043
≥ 65 years	19.77%	20.41%		20.13%	20.30%	
Gender			0.0162			− 0.0008
Female	42.64%	41.84%		41.95%	41.99%	
History of comorbidity						
Hypertension	58.33%	57.34%	0.0199	57.38%	57.52%	− 0.0029
Hyperlipidemia	59.76%	56.96%	0.0567	57.51%	57.44%	0.0014
Ischemic heart disease	2.57%	2.02%	0.0386	2.10%	2.11%	− 0.0006
Heart valve surgery	0.09%	0.05%	0.0200	0.05%	0.05%	− 0.0032
Obstructive sleep apnea	0.00%	0.00%		0.00%	0.00%	
Hyperthyroidism	2.68%	2.08%	0.0406	2.19%	2.19%	0.0003
Chronic kidney disease	7.56%	7.09%	0.0182	7.11%	7.17%	− 0.0024
PAOD	0.51%	0.36%	0.0239	0.36%	0.39%	− 0.0038
Gout	19.31%	20.61%	− 0.0323	20.37%	20.39%	− 0.0006
Chronic lung disease	1.29%	1.35%	− 0.0049	1.31%	1.34%	− 0.0026
Congestive heart failure	0.28%	0.13%	0.0355	0.15%	0.16%	− 0.0015
Medication						
Beta-blocker	13.78%	13.75%	0.0008	13.67%	13.75%	− 0.0026
Diltiazem/verapamil	3.02%	2.38%	0.0408	2.48%	2.49%	− 0.0002
Statin	33.60%	25.83%	0.1745	27.18%	27.20%	− 0.0005
ACEI/ARB	36.62%	30.55%	0.1305	31.66%	31.61%	0.0012

*ACEI* angiotensin-converting-enzyme inhibitor, *AF* atrial fibrillation, *ARB* angiotensin II receptor antagonists, *CI* confidence interval, *DM* diabetes mellitus, *DPP4i* dipeptidyl peptidase-4 inhibitor, *GLP-1* glucagon-like peptide-1, *PAOD* peripheral arterial obstructive disease, *TZD* thiazolidinedione

**Fig. 2:**
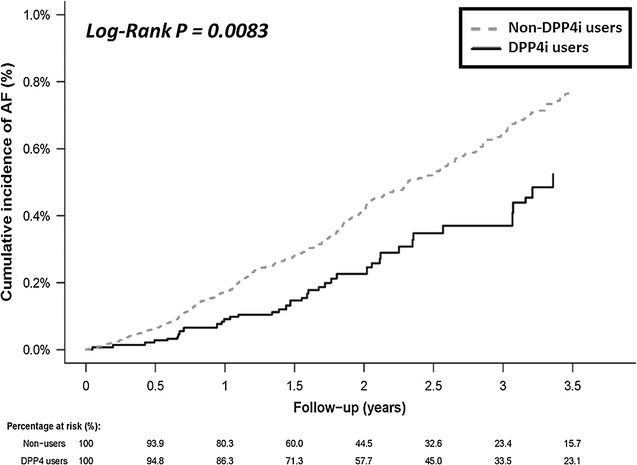
Cumulative risk curve of new-onset AF for the study cohorts treated with metformin plus DPP-4 inhibitor versus other hypoglycemic agents after propensity score weighting. DPP4i group (solid line) shows a significantly lower cumulative risk of new-onset AF compared with non-DPP4i group in patients treated with metformin (dotted line). *DPP4i* dipeptidyl peptidase-4 inhibitor

**Fig. 3:**
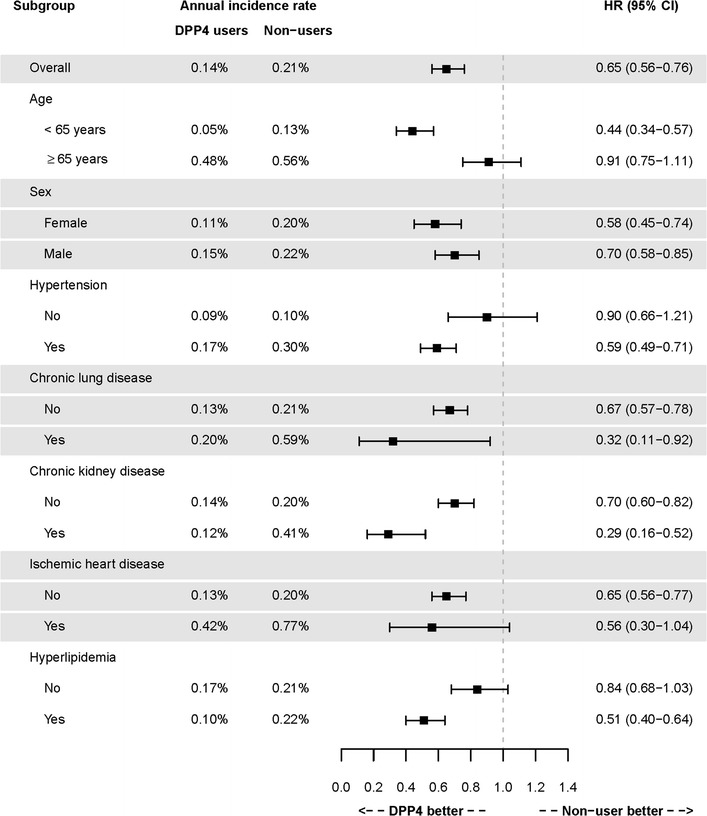
Forest plot of hazard ratio of risk of new-onset AF for DM patients treated with metformin plus DPP-4 inhibitor versus other hypoglycemic agents after propensity score weighting. DPP4i is shown to be associated with a lower risk of new-onset AF compared with other hypoglycemic agents in most subgroups. *DPP4i* dipeptidyl peptidase-4 inhibitor

DPP4i users were associated with a lower risk of new-onset AF compared with non-DPP4i users, either before or after propensity-score weighting [hazard ratio (HR): 0.65; 95% confidential interval (CI) 0.56–0.76; *P* < 0.0001]. It was noted that most HAs, with the exception of insulin/GLP1, were associated with a significantly higher risk of new-onset AF when compared with DPP4i (Table 2). Figure 2 and Additional file 2: Figure S1 show a clear separation of event curves for new-onset AF between these two groups either before or after propensity score weighting adjustment. The time interval from the index day to the occurrence of AF in DPP4i user vs non-user is 1.9 ± 2.9 years and 1.7 ± 1.2 years respectively. Some patients did not take DPP4i at the diagnosis of the first AF. Among the 45 AF events in DPP4i users, 10 of them (22.2%) did not take DPP4i within 3 months of the events. Subgroup analysis revealed that DPP4i usage was associated with a lower risk of new-onset AF compared with non-DPP4i usage in most subgroups (Fig. 3).

**Table 2:** Incidence (per 100 person-years) of new-onset AF in diabetic patients taking metformin plus DPP4i or other hypoglycemic agents

	New-onset AF				
	Numbers	Events	Incidence before weighting	Incidence after weighting	HR^a^ 95% CI P value
DPP4i	16,017	45	0.14 (0.10–0.18)	0.14 (0.12–0.15)*	1.00 (Reference)
Other hypoglycemic agents rather than DPP4i	74,863	386	0.21 (0.19–0.24)	0.21 (0.19–0.23)	1.53 (1.31–1.78) P < 0.0001
Sulfonylurea	62,216	318	0.20 (0.18–0.23)	0.20 (0.18–0.23)	1.45 (1.24–1.70) P < 0.0001
Alpha glucosidase inhibitor	5091	24	0.24 (0.14–0.33)	0.23 (0.15–0.32)	1.75 (1.19–2.57) P = 0.0045
Meglitinide	5164	41	0.38 (0.26–0.49)	0.38 (0.27–0.48)	2.78 (2.05–3.76) P < 0.0001
TZD	3091	15	0.23 (0.11–0.34)	0.23 (0.12–0.33)	1.68 (1.05–2.69) P = 0.0307
Insulin	1361	3	0.11 (0.02–0.32)	0.11 (0.03–0.29)	0.79 (0.28–2.26) P = 0.20
GLP1	5	0			

There were 2016 patients taking more than two hypoglycemic agents as second-line hypoglycemic agents at the same time

*ACEI* angiotensin-converting-enzyme inhibitor, *AF* atrial fibrillation, *ARB* angiotensin II receptor antagonists, *CI* confidence interval, *DM* diabetes mellitus, *DPP4i* dipeptidyl peptidase-4 inhibitor, *GLP-1* glucagon-like peptide-1; *PAOD* peripheral arterial obstructive disease, *TZD* thiazolidinedione

^a^For other hypoglycemic agents versus DPP-4 inhibitors (reference) after propensity score weighting

In Table 3, Cox’s model was performed after propensity score weighting in order to identify the independent risk factors for the new-onset AF for those patients taking HAs. The multivariate analysis indicated that use of DPP4i was associated with lower risk of new-onset AF (HR 0.69; 95% CI 0.59–0.81; *P* < 0.0001), and age > 65 years (HR 4.75; 95% CI 4.07–5.55; *P* < 0.0001), presence of hypertension (HR 1.74; 95% CI 1.45–2.06; *P* < 0.0001), and ischemic heart disease (HR 1.98; 95% CI 1.48–2.66; *P* < 0.0001) were independent risk factors for new-onset AF.

**Table 3:** Predictors of new-onset AF for diabetic patients taking hypoglycemic agents after propensity score weighting

	Hazard ratio (95% CI); P value	
	Univariate	Multivariate
DPP4i versus other hypoglycemic agents	0.65 (0.56–0.76); < 0.0001	0.69 (0.59–0.81); P < 0.0001
Age (years)		
< 65	1.00 (reference)	1.00 (reference)
≥ 65	5.76 (4.97–6.68); < 0.0001	4.75 (4.07–5.55); P < 0.0001
Female gender	0.87 (0.75–1.01); 0.0714	
Chronic lung disease	2.28 (1.46–3.56); 0.0003	
Chronic kidney disease	1.74 (1.37–2.21); < 0.0001	
Hypertension	2.64 (2.22–3.13); < 0.0001	1.74 (1.45–2.06); P < 0.0001
Ischemic heart disease	3.84 (2.88–5.13); < 0.0001	1.98 (1.48–2.66); P < 0.0001

*ACEI* angiotensin-converting-enzyme inhibitor, *AF* atrial fibrillation, *ARB* angiotensin II receptor antagonists, *DM* diabetes mellitus, *DPP4i* dipeptidyl peptidase-4 inhibitor, *GLP-1* glucagon-like peptide-1, *PAOD* peripheral arterial obstructive disease, *TZD* thiazolidinedione

## Discussion

The nationwide cohort study evaluated the risk of new-onset AF in metformin-based patients with diabetes taking DPP4i versus other second-line hypoglycemic agents. Recently we had presented metformin users were associated with a lower risk of AF in patients with diabetes compared with non-users [20, 21]. In this study, in addition to use of metformin, we observed that patients taking DPP4i had a significantly lower risk of new-onset AF than those treated with other HAs including sulfonylurea, alpha-glucosidase inhibitors, meglitinide, and thiazolidinedione. The risk reduction of new-onset AF for DPP4i users versus other HAs was similar among most subgroups.

Several studies have indicated that diabetes, as well as age, hypertension, and structural heart diseases, are independent risk factors for AF [22]. Atrial fibrosis and chronic inflammation are known to contribute to AF [23, 24]. Diabetes is associated with numerous metabolic defects which could be responsible for AF occurrence. Diabetes could also cause structural, electrical, electromechanical and autonomic remodeling, triggering AF in patients with diabetes [25]. In several animal studies, DPP4i inhibitors increase in threshold of ventricular fibrillation during the ischemic period and stabilized cardiac electrophysiology, protected cardiac mitochondrial function [26]. DPP4i is commonly used for the treatment of patients with diabetes in clinical practice. By inhibiting the degradation of GLP-1, DPP4i has been shown to increase the serum levels of GLP-1, which indirectly stimulate insulin secretion and enhance beta-cell function. DPP-4 is highly expressed in endothelial cells and the GLP-1 receptor is expressed on cardiomyocytes, vascular smooth muscle cells, and endothelial cells. A previous animal study showed that DPP4i had both GLP-1-dependent and GLP-1-independent cardioprotective effects using an ischemic heart model [27]. A recent study also showed that DPP4i may exert antiarrhythmic effects and reduce infarct size during myocardial ischemia and reperfusion [28]. There were several mechanisms to explain the relationship between diabetes and AF and the mechanism of DPP4i in lowering AF risk. Recently Chang et al. showed that in spontaneously hypertensive rats, sitagliptin would modulate the electrical and mechanical properties of pulmonary veins and atria, suggesting that DPP4i may be protective against AF genesis [29]. Furthermore, Yamamoto et al. [13]. demonstrated that alogliptin, a DPP4i, can shorten the AF duration caused by ventricular tachy-pacing in rabbits with fibrotic atria. The underlying mechanisms may include augmentation of atrial remodeling and improvement of mitochondrial function [30]. Further, administration of nitric oxide synthase inhibitor has been shown to block the protective effects of alogliptin via shortening AF duration, capillary density, and atrial fibrosis. Their findings suggest that DPP4i may have an antiarrhythmic effect in the prevention of heart-failure-induced AF [13]. In contrast, Hayami et al. [31]. demonstrated that administration of sulfonylurea and DPP4i both inhibited inflammation and fibrosis of the atria in streptozotocin-induced diabetic rats. However, no significant differences were observed between the two oral HAs. They concluded that reduced atrial fibrosis may derive from the tight control of blood glucose levels rather than a drug-specific anti-inflammatory property.

The monotherapy of metformin is suggested as first-line therapy for glycemic control in newly diagnosed patients with type 2 diabetes according to the current guidelines [32]. If monotherapy does not achieve the therapeutic goal, a second HA would be added [33]. Several studies have indicated that patients with diabetes have an increased risk of developing adverse cardiovascular outcomes [34]. Therefore, prevention of any adverse cardiovascular outcome seems to be an important consideration when choosing second-line HAs. Until now, only empagliflozin, a potent inhibitor of sodium glucose cotransporter 2 (SGLT2), and metformin have provided cardioprotective effects in patients with diabetes beyond the hypoglycemic effects [35, 36]. It is unclear if GLP-1 receptor agonists was associated with AF [37]. At the present time, it is still unclear whether DPP4i would lead to better outcomes with a reduction in incidence of major adverse cardiovascular events [38–41]. Recent large scale clinical trials, including EXAMINE, SAVOR-TIMI53, and TECOS, all failed to show significant improvement in cardiovascular outcomes in type 2 diabetic patients treated with DPP4i [38, 42, 43]. In contrast, several nationwide cohort studies presented that DPP4i may have cardioprotective properties, including the ability to lower the incidence of heart failure, coronary heart disease, and stroke [15, 39, 44]. In subgroup analysis (Fig. 3), however, we did not find differences in the occurrence of new-onset AF between DPP4 users and non-users in patients > 65 years. It is likely that more AF-precipitating factors coexist in aged diabetic patients, which would remove the protective effect of DPP4i. Until now, no randomized control trials directly compared the risk of new-onset AF in patients with diabetes taking DPP4i compared with patients taking other HAs. Our data revealed that DPP4i was associated with a lower risk of new-onset AF in patients with diabetes, indicating that DPP4i as a feasible second-line oral HA for AF primary prevention. Future prospective studies are necessary, however, to evaluate the potential role of DPP4i on atrial remodeling and AF prevention in the setting of diabetes.

### Study limitations

Our study had several limitations including lack of laboratory data such as hemoglobin A1c levels, blood sugar levels, renal function, and lipid profiles, thus, the severity of diabetes in each patient could not be classified. Furthermore, because of the lack of EKG data, the contribution of persistent AF or paroxysmal AF to acute illness could not be assessed. In addition, although an extensive number of variables had been selected for our propensity score model, and a close balance among those factors was successfully achieved in our study, there were other unmeasured confounding factors that may have biased our results including the use of tobacco and/or alcohol, body mass index, family history, and physicians’ preference for a specific HA. Also, coding errors regarding outcomes and comorbidities may have existed because of each physician’s different response when caring for their own patients. Finally, this was a retrospective, observational study. Therefore, further prospective randomized studies are needed to determine whether our findings are applicable to non-Asian patients with type 2 diabetes.

## Conclusions

Among patients with diabetes prescribed with metformin, the patients with DPP4i as second HA were associated with a lower risk of AF compared with the patients with other drugs as second HAs in real-world practice.

## Additional files

**Additional file 1: Table S1.** International Classification of Disease (9th edition) Clinical Modification (ICD 9-CM) codes used to define the co-morbidities and clinical outcome in the study cohort.

**Additional file 2: Figure S1.** Cumulative risk curve of the new-onset AF for the study cohorts treated with metformin plus DDP-4 inhibitor or other hypoglycemic agents before propensity score weighting. DDP4i group (solid line) had a significantly lower cumulative risk of new-onset AF compared with non-DDP4i group in patients treated with metformin (dotted line). *DPP4i* dipeptidyl peptidase-4 inhibitor.

## Abbreviations

AF: atrial fibrillation
ASMD: absolute standardized mean difference
DM: diabetes mellitus
DPP4i: dipeptidyl peptidase-4 inhibitor
HA: hypoglycemic agents
ICD-9-CM: International Classification of Diseases, ninth revision, Clinical Modification
IPTW: inverse probability of treatment weighting
RCT: randomized clinical trial
LHDB: Longitudinal Cohort of Diabetes Patients Database
NHIRD: National Health Insurance Research Database
