# Supplementary material for: Dipeptidyl peptidase-4 inhibitor decreases the risk of atrial fibrillation in patients with type 2 diabetes: a nationwide cohort study in Taiwan
Source: Cardiovasc Diabetol. 2017 Dec 19;16:159. doi: 10.1186/s12933-017-0640-5 (PMC5735601; doi:10.1186/s12933-017-0640-5)
Supplement: Supplementary file 1 — Additional file 1: Table S1. International Classification of Disease (9th edition) Clinical Modification (ICD 9-CM) codes used to define the co-morbidities and clinical outcome in the study cohort. [file 12933_2017_640_MOESM1_ESM.doc]

**Additional Materials**

**Table S1**

International Classification of Disease (9^th^ edition) Clinical Modification (ICD 9-CM) codes used to define the co-morbidities and clinical outcome in the study cohort

| Disease | ICD-9 Codes | Diagnosis definition |
| --- | --- | --- |
| **Major adverse cardiovascular events (MACE)** | | |
| Ischemic stroke | 433, 434, 436 | Discharge primary code |
| [Transient ischemic attack](http://en.wikipedia.org/wiki/Transient_ischemic_attack) | 435 | Discharge primary code |
| Myocardial infarction | 410, 411, 412 | Discharge primary code |
| Congestive heart failure | 428 | Discharge primary code |
| Malignant arrhythmia | 426.0, 426.12–426.13, 426.51–426.52, 426.54, 427.1, 427.4, 427.41–427.42, 427.5 | Discharge primary code |
| Cardiogenic shock | 785.51 | Discharge primary code |
| **Primary outcome** | | |
| Atrial fibrillation | 427.31 | Discharge or outpatient department ≥1 |
| **Co-morbidities** | | |
| Ischemic heart disease | 410-414 | Outpatient department ≥2 |
| Peripheral arterial occlusive disease | 440.2 | Outpatient department ≥2 |
| Hypertension | 401, 402 | Outpatient department ≥2 |
| Diabetes mellitus | 250 | Outpatient department ≥2 |
| Hyperlipidemia | 272 | Outpatient department ≥2 |
| Gout | 274 | Outpatient department ≥2 |
| Hyperthyroidism | 242 | Outpatient department ≥2 |
| Chronic kidney disease | 580-589 | Outpatient department ≥2 |
| Obstructive sleep apnea | 327.23 | Outpatient department ≥2 |
| Chronic lung disease | 490-494, 495.8, 495.9, 496, 500, 502-505 | Discharge |
