# Supplementary figures and images for: Dipeptidyl peptidase-4 inhibitor decreases the risk of atrial fibrillation in patients with type 2 diabetes: a nationwide cohort study in Taiwan
Source: Cardiovasc Diabetol. 2017 Dec 19;16:159. doi: 10.1186/s12933-017-0640-5 (PMC5735601; doi:10.1186/s12933-017-0640-5)

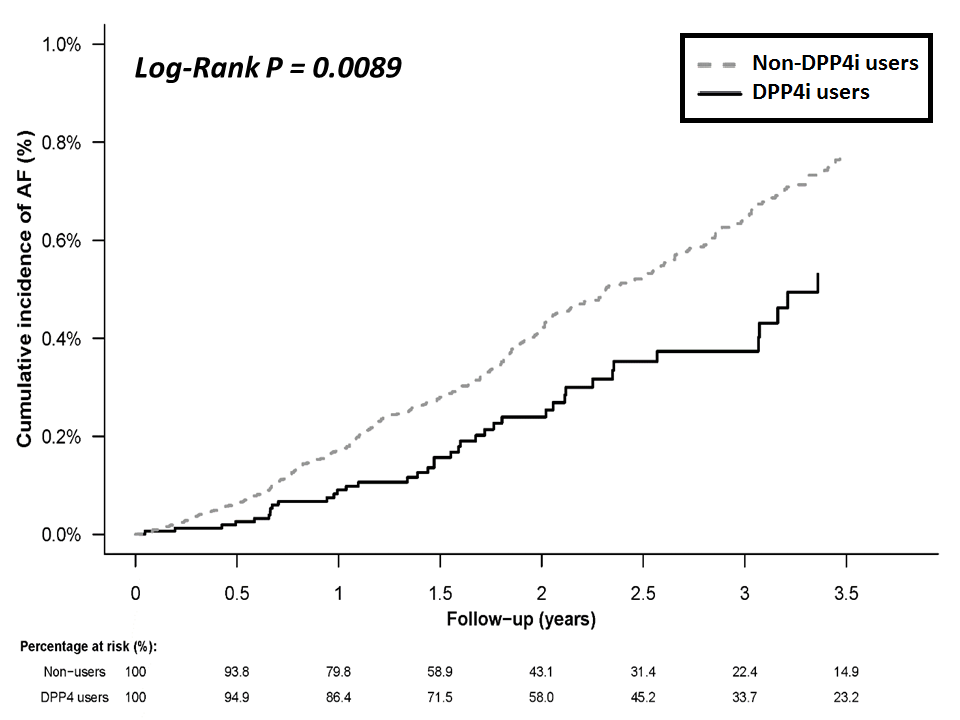

Supplement: Supplementary file 2 — Additional file 2: Figure S1. Cumulative risk curve of the new-onset AF for the study cohorts treated with metformin plus DDP-4 inhibitor or other hypoglycemic agents before propensity score weighting. DDP4i group (solid line) had a significantly lower cumulative risk of new-onset AF compared with non-DDP4i group in patients treated with metformin (dotted line). DPP4i dipeptidyl peptidase-4 inhibitor. [file 12933_2017_640_MOESM2_ESM.tif]
